# Analysis of Food Labels to Evaluate the Nutritional Quality of Bread Products and Substitutes Sold in Italy: Results from the Food Labelling of Italian Products (FLIP) Study

**DOI:** 10.3390/foods9121905

**Published:** 2020-12-20

**Authors:** Donato Angelino, Alice Rosi, Emilia Ruggiero, Daniele Nucci, Gaetana Paolella, Veronica Pignone, Nicoletta Pellegrini, Daniela Martini, on behalf of the SINU Young Working Group

**Affiliations:** 1 Faculty of Bioscience and Technology for Food, Agriculture and Environment, University of Teramo, 64100 Teramo, Italy; dangelino@unite.it; 2 Human Nutrition Unit, Department of Food and Drug, University of Parma, 43121 Parma, Italy; alice.rosi@unipr.it; 3 Department of Epidemiology and Prevention, IRCCS Neuromed, 86077 Pozzilli, Italy; emilia.ruggiero@moli-sani.org; 4 Digestive Endoscopy Unit, Veneto Institute of Oncology IOV-IRCCS, 35100 Padua, Italy; daniele.nucci@iov.veneto.it; 5 Department of Chemistry and Biology A. Zambelli, University of Salerno, 84084 Fisciano, Italy; gpaolella@unisa.it; 6 Freelance Nutritionist, 82018 San Giorgio del Sannio, Italy; veropign@hotmail.it; 7 Department of Agricultural, Food, Environmental and Animal Sciences, University of Udine, 33100 Udine, Italy; 8 Department of Food, Environmental and Nutritional Sciences (DeFENS), Università degli Studi di Milano, 20133 Milan, Italy; Daniela.martini@unimi.it

**Keywords:** bread, bread substitutes, food labeling, nutritional quality, gluten free, nutrition and health claims

## Abstract

Bread is one of the most common staple foods, despite the increasing consumption of the so-called “bread substitutes”. The aim of the present work is to survey the nutritional quality intended as a nutrition declaration of 339 pre-packed bread products and 1020 bread substitutes sold in the major retailers present on the Italian market. Comparisons of energy, macronutrient, and salt content within product types, and between regular and gluten-free (GF) products and products with or without nutrition claim (NC) and health claim (HC) declarations, were performed. A high inter-product variability was detected. The median energy contents were 274 (interquartile range 255–289) and 412 (380–437) kcal/100 for bread products and substitutes, respectively. Irrespective of the category, GF products had lower amounts of energy than their gluten-containing counterpart (*p* < 0.001), whereas products carrying NC had lower energy, sugar and salt amounts than the products without these declarations on the pack (*p* < 0.001 for all). A strong positive correlation was observed between energy and carbohydrate in bread (rho = 0.73, *p* < 0.001), but not in substitutes (rho = 0.033, *p* = 0.29). The present work highlighted a high variability in the apparent nutritional quality of bread products and substitutes sold on the Italian market, and suggested that bread alternatives should not be considered *tout court* as substitutes from a nutritional point of view.

## 1. Introduction

Bread is one of the most consumed cereal-based staple foods, with an estimated consumption of about 100 g per day in Italy and in other European countries [1,2]. Bread is rich in carbohydrate, is a good source of protein and plays an important role in daily energy intake [3]. It is also a good source of vitamins (mainly from the B-vitamin group) and minerals (such as iron, calcium, phosphorus, zinc, potassium, and magnesium) [3].

Nevertheless, bread consumption has continued to decline mostly in European countries, even though the European market accounts for over 45% of the global consumption by volume [4]. The decline in bread consumption might be due to the general public belief that “bread fattens” [5,6], or to its high glycemic index compared to other cereal-based products, such as pasta [7,8]. However, it is worth pointing out that a correlation between bread consumption and weight gain has not been established so far [4,8,9].

Other considerable points for its decreasing consumption are the perception of a higher price compared to the past and uncertainty of its nutritional value [10]. Concerning this last point, it has been evidenced that the intention-to-buy of consumers, the perception of sensory intensities, and the health- and nutrition-related attributes may also be influenced by the nutrition information provided on the food pack [11]. Among these attributes, the presence of a gluten-free (GF) declaration has been shown to be one of the most important determinants of food choices, not only by celiac disease patients [12], but it also affects the intention-to-buy of non-celiac people [13]. Similarly, products with nutrition and health claims (NHC) may be perceived as healthier compared with products not boasting claims, and this may affect food choice [14] and may increase food consumption and overall energy intake [15]. Therefore, food labeling is one of the most important means of communicating relevant information to consumers about the product’s identity and content, as well as driving the buying decisions of the customers. In Europe, the provision of food information to consumers is mostly regulated by the Council Regulation (EU) 1169/2011, which establishes the mandatory food information [16]. Besides this information, many other details can be conveyed, including NHC as regulated by the Council Regulation (EC) 1924/2006, which generally refer to one or more nutrients and therefore are not indicative of the overall quality of a food [17]. It is particularly worthy of consideration for the cereal-based food category the European Implementing Regulation (EU) No. 828/2014, which regulates the information concerning the absence or reduced presence of gluten in food [18].

In recent years, a number of studies have investigated how consumers perceive and use nutrition labels, and have assessed consumer preferences for different nutrition labeling schemes [19,20,21]. Nevertheless, only a few studies with contrasting results have investigated whether the presence of specific information on the food pack can be considered a marker of the overall quality of food products. For instance, Taillie and colleagues examined the nutrition claims and nutritional quality of more than 80 million food and beverages purchased in the United States, highlighting that foods bearing low-content claims did not necessarily have better overall nutritional profiles compared to products without claims [22]. Similarly, a survey performed in Brazil on over 5600 packaged foods evidenced that children’s products with nutrient claims failed in evidencing a significantly improved nutrition profile compared to counterparts without nutrient claims [23]. On the contrary, a Canadian survey on more than 15,000 food and beverages found an overall better nutritional profile, in terms of the fewer calories, and less saturated fat, sodium, and sugar, and higher contents of protein and fiber, of products carrying nutrition claims compared to products without them [24].

In this scenario, to broaden the knowledge on the relation between information on the food pack and the overall nutritional quality of products, on 2018 the Food Labelling of Italian Products (FLIP) project was conceived by the Working Group SINU (Italian Society of Human Nutrition) Young, with the aim of systematically evaluating the nutritional quality of commercial foods sold on the Italian market. The aim of the present work was to investigate the nutritional declaration of pre-packed bread and bread substitute categories mainly sold in Italy, by collecting their nutritional information from their food label. Then, we aimed at comparing differences in energy and nutrient content within bread and bread substitute categories, whereby products were classified for different characteristics: type of product, GF declaration and presence of NHC.

## 2. Materials and Methods

### 2.1. Product Selection

The survey was designed as a comprehensive analysis of all bread products and substitutes sold by 13 out of the major retailers present on the Italian market, and allowing purchases in the e-commerce mode (Auchan, Bennet, Carrefour, Conad, Coop Italia, Crai, Despar, Esselunga, Il Gigante, Iper, Pam Panorama, Selex, Sidis). The online search was conducted from July 2018 until December 2018. The inclusion/exclusion criteria and the collected information have been previously extensively described [25]. Briefly, the inclusion criteria for products were to be breads and bread substitutes present in at least one online shop during the collection period. Products were excluded from the survey if they were not pre-packed, or in cases of incomplete images of all the sides of the pack or of unclear images of the nutrition declaration or list of ingredients. Finally, products marked as ‘product currently unavailable’ on all the online stores during the collection period were not considered.

### 2.2. Data Collection

Briefly, for each product the contents of energy (kcal/100 g), protein (g/100 g), carbohydrate (g/100 g), sugars (g/100 g), total fat (g/100 g), saturates (g/100 g) and salt (g/100 g) were recorded from food labels, together with other information such as product name, brand name, descriptive name and voluntary declarations including NHC (i.e., presence or absence of the least one nutrition or heath claim) and GF statement (either “specifically formulated for celiac”, or “containing gluten”). The precision of the extracted data was double-checked by two independent researchers and disagreements were solved through secondary extractions with the help of a third researcher.

The collected data were organized in a dataset in which items were grouped for: category (bread products and substitutes); gluten free (yes/no); nutrition claim (yes/no) and health claim (yes/no). Further, based on descriptive name, the products of each category were sub-grouped as the following types: (A) loaf, (B) rolls and (C) sliced bread, within the bread category; and (D) crackers, (E) wraps, (F) breadsticks, (G) rice and corn cakes, (H) taralli, (I) croutons, bruschetta and “frisella”-bread and (J) rusks, within the bread substitute category.

### 2.3. Statistical Analysis

Statistical analyses were carried out using IBM SPSS Statistics for Macintosh, Version 26.0. Armonk, NY: IBM Corp, setting the significance level at *p* < 0.05. Variables were expressed as median and interquartile range. The normal distribution of data was rejected using the Kolmogorov–Smirnov test. Differences in energy and nutrient contents per 100 g among product types were assessed using the Kruskal–Wallis non-parametric one-way ANOVA for independent samples with multiple pairwise comparisons, whereas differences between GF declaration categories, nutrition claim categories, and health claim categories were analyzed using the Mann–Whitney non-parametric test for two independent samples. Product variability between the two categories (bread products vs. substitutes) and among types within each product category was assessed by using a principal component analysis (PCA) with varimax rotation, considering energy and nutrient contents per 100 g of products.

Last, a Spearman’s correlation test was performed to explore the bivariate correlation between energy density (kcal/100 g) and total fat (g/100 g), energy density (kcal/100 g) and carbohydrate (g/100 g), and total fat (g/100 g) and carbohydrate (g/100 g) within each product category.

## 3. Results

A total of 343 bread items and 1173 bread substitutes were initially identified during the research conducted in the online stores. Among these, 339 and 1020 bread products and bread substitutes, respectively, matched the inclusion criteria and were considered for analysis (Table 1 and Table 2). Thus, about 90% of the products sold in the considered retailers were retrieved. The inter-rater agreement in the excluding of products was 97%, and the remaining 3% of disagreements was successfully resolved by a third researcher.

Among bread products, ~50% of the items were sliced bread, followed by loaf and rolls that accounted for 30% and 20%, respectively. Conversely, among bread substitutes, the most numerous were breadsticks and crackers, accounting for about 19% and 18%, respectively, followed by rice and corn cakes (~17%), wraps (~14%), rusks (11%) and finally, taralli and “Croutons, Bruschetta and Frisella” bread (both ~10%).

GF products accounted for less than 15% of the total number for both categories. By considering the presence of NHC on the food package, about 27% and 35% of the total products for bread items and substitutes, respectively, contained at least one nutrition claim, while no bread items and only ~5% substitutes contained at least one health claim.

### 3.1. Nutritional Composition of Bread Products and Substitutes

The nutritional composition of the retrieved bread food labels is reported in Table 1. Overall, the median energy content was 274 (255–289) kcal/100 g, and this was higher in rolls compared to both loaf and sliced bread. Rolls contained also the highest amount of total fat, saturates and sugars. Total carbohydrates were similar in loaf and rolls but significantly higher than sliced bread products, and protein and salt contents were similar among all bread types. GF bread types were characterized by lower energy, carbohydrate, sugars, and protein contents and by higher total fat and saturates amounts than their gluten counterparts. No statistical differences in salt content were found. Bread products with at least one nutrition claim were characterized by a lower energy, saturates, carbohydrate, sugars and salt content compared to products without nutrition claims.

The nutritional composition of bread substitutes is shown in Table 2. The median energy content was 412 (380–437) kcal/100 g, almost double that of the energy content of bread products. Taralli had the highest energy, total fat, saturates and salt contents, rice and corn cakes had the highest carbohydrate amount and the lowest total fat and saturates, sugars, and salt contents. Wraps had the lowest energy, total carbohydrate, and protein amounts, while rusks had the highest sugars contents, and breadsticks had the highest protein amount. GF bread substitutes had lower amounts of energy and nutrients than gluten-containing products, except for total carbohydrates which were higher in GF products. Similarly, bread substitutes with at least one nutrition claim had lower energy and nutrients contents, except total carbohydrates and proteins, than products without nutrition claims. No differences were found among energy content in bread substitutes with and without health claims, despite the fact that the former had lower total fat and saturates contents but a higher protein content.

### 3.2. Variability of the Nutritional Composition of Bread Products and Substitutes

**Figure 1:**
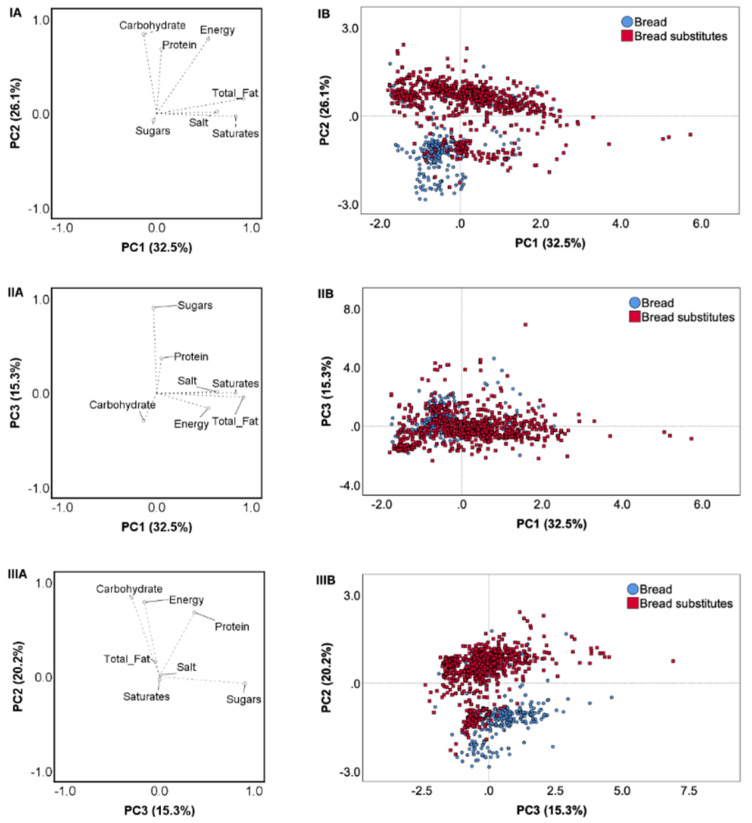
Principal component analysis (PCA) describing the inter-category variability based on the nutritional composition of analyzed products (energy (kcal/100 g), total fat (g/100 g), saturates (g/100), carbohydrate (g/100 g), sugars (g/100 g), protein (g/100 g), and salt (g/100 g)). Loading plots of PC1 versus PC2 (IA), PC1 versus PC3 (IIA) and PC3 versus PC2 (IIIA); score plots of the nutrition composition for bread and bread substitutes of PC1 versus PC2 (IB), PC1 versus PC3 (IIB) and PC3 versus PC2 (IIIB).

The nutritional profile of bread products and bread substitutes was explained by three principal components (PCs), which described 73.9% of the total variability (Figure 1). PC1 had a high loading on total fat, saturates and salt, while PC2 was characterized by high values of carbohydrates and protein. Energy content was a high-loading component in both PC1 and PC2. The third component (PC3) loaded highly on sugars. Although a high inter-product variability was observed among products belonging to both bread items and bread substitutes, they were clustered along the PC2 axis, energy, carbohydrate and protein contents being the main discriminatory components between the two product categories.

A high inter-product variability was also observed when bread and bread substitute types were analyzed separately. Three PCs explained 75.5% of the total variability among bread products (Supplementary Materials Figure S1). The first component had high loadings on energy, carbohydrate and protein contents. Total fat, saturates and sugars loaded PC2, while salt was the main loading component of PC3. Within bread types, sliced bread products clustered along the three PCs, while loaf and roll products presented a higher variance of nutrient values. The variability among bread substitutes types (Figure S2) was explained for 71.0% by three PCs. The first PC was characterized by high values of total fat, saturates and energy, PC2 by high contents of carbohydrates, and PC3 by high protein and sugars amounts. Salt was a positive loading factor for PC1, while it was a negative component on PC2. Products belonging to wraps and to rice and corn cakes clustered mainly on PC2, based on their low and high carbohydrate contents, respectively. The other bread substitute types were similar and showed great variance in their energy and nutrient contents.

### 3.3. Bivariate Correlations among Energy and Macronutrients

**Figure 2:**
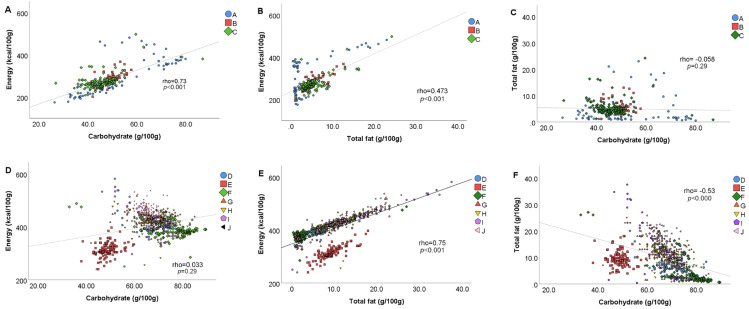
Bivariate correlations between energy (kcal/100 g), total fat (g/100 g) and carbohydrates (g/100 g) contents of the analyzed bread and bread substitutes. Plots are relative to correlation between energy and carbohydrate contents in bread (**A**) and bread substitutes (**D**), energy and total fat contents in bread (**B**) and bread substitutes (**E**) and total fat and carbohydrate contents in bread (**C**) and bread substitute (**F**) items. Legend: A, loaf; B, rolls; C, sliced bread. D, crackers; E, wraps; F, breadsticks; G, rice and corn cakes; H, taralli; I, croutons, bruschetta and “Frisella” bread; J, rusks.

Bivariate correlations among energy and macronutrient contents, in both bread products and bread substitutes, are shown in Figure 2. In the former, a strong positive correlation was found between carbohydrate and energy content (rho = 0.73, *p* < 0.001, Figure 2A), and a moderate positive correlation was found between total fat and energy contents (rho = 0.47, *p* < 0.001, Figure 2B), while no correlation was observed between carbohydrate and total fat contents (rho = −0.058, *p* = 0.29, Figure 2C). Regarding bread substitutes, carbohydrate and energy contents were not correlated (rho = 0.033, *p* = 0.29, Figure 2D), while a strong positive correlation emerged between total fat and energy contents (rho = 0.75, *p* < 0.001, Figure 2E) and a moderate negative correlation was found between carbohydrate and total fat contents (rho = −0.53, *p* < 0.001, Figure 2F).

## 4. Discussion

In the present study, more than 1300 pre-packed bread products and bread substitutes, currently sold in Italy, have been evaluated for their apparent nutritional quality, stated by their nutritional declaration, by retrieving the information present on the food pack. Two major aspects emerged from this survey: the elevated number of items present on the market and the high variability of nutritional values found among the considered products. Although in Italy it is still common to buy fresh baked and unpacked bread (here not considered because the nutrition declaration is not mandatory based on the Council Regulation (EU) No. 1169/2011 [16]), more than three hundred bread items and more than one thousand items for bread substitutes have been analyzed. Mostly for the last category, it is clear that the wide offering of products and their ease of use—being pre-packed, with a long shelf-life and often in a single serving—increased the consumer attention, as suggested by the increasing sales, with a trade of EUR 400 million in 2019 [26].

Energy, macronutrient and salt values were very similar among the three different types of bread products, with significant but slightly higher values of energy, total fat, saturates and sugars for rolls compared to loaf and sliced bread types. Regarding sliced bread products, they showed a low variability in terms of nutrient content, suggesting a high homogeneity in terms of formulation and ingredients used. On the contrary, differently from the bread category, where products showed few significant differences for the energy and nutrient content, bread substitutes evidenced wider inter-product variability, as highlighted by the PCA. In particular, taralli showed the lowest apparent nutritional quality, with higher energy, fats and salt and lower carbohydrates and proteins compared to the other categories. This difference is probably due to the low moisture in taralli and its different formulation, which is richer in fats than other items. When compared with other surveys focusing on Italian products, the data are substantially in agreement for both bread and substitute items, despite the fact that there are no available data split into the different types [27].

Intriguing results emerged when the contents of some macronutrients in bread and bread substitutes were correlated with their total energy. As expected for bread products, a strongly significant positive correlation has been found between carbohydrates and energy. Conversely, in bread substitutes, energy was strongly positively correlated with total fat. This means that the so-called “bread substitutes” have a lower apparent nutritional quality than the products they would substitute. In detail, these products have a different formulation in terms of ingredients compared to bread, and a higher content of energy, total fat, and saturates, due to a lower water content. Even wraps show a higher fat content compared to bread, despite a similar moisture content (that can be deduced considering the macronutrient contents). Thus, the putative replacement of the suggested daily portion of bread with such bread substitutes might decrease the energy intake from available carbohydrates—which in Italy barely reaches 45% of the total daily energy intake—and, on the other hand, might increase the energy intake from fat—which in Italy is over 35% of the total daily energy intake [28]. Therefore, due to the highlighted nutritional differences, such products cannot be considered as substitutes of bread. Moreover, the different types of bread substitutes widely vary for their nutrient contents and, thus, cannot be considered equivalent from a nutritional point of view. Nutritional differences between bread and substitutes were partially evidenced in the last edition of the Italian Dietary Guidelines, where different frequencies of consumption were suggested for the two categories of products (i.e., 3.5 portions/day and 1 portion/week for bread and substitutes, respectively, for a daily energy intake of 2000 kcal) [29]. On the whole, these results support the importance of a wide dissemination of the Food-Based Dietary Guidelines among the general population to help consumers in making informed and possibly healthy food choices.

The analysis of the food labels of GF bread products evidenced global lower energy, carbohydrates and protein amounts compared to their counterparts. Except for the higher total carbohydrate content, GF bread substitutes reported the same results, but also a lower fat and salt content compared to their gluten-containing counterparts. Contrasting results have been found in the literature, where different international surveys have been published. Our data partially confirm those of a Spanish survey on GF bread items vs. gluten-containing ones [30], where the GF bread category had lower energy and protein amounts as well as higher total fat and saturates than their counterparts. Despite similarities with the Spanish survey [30], the GF bread items retrieved in our study had overall lower amounts of both total fat and saturates than the Spanish ones. Three recent surveys retrieved data from food labels of regular and GF white and brown bread types sold on the UK market. From a sample of more than 1570 items, Fry et al. found a significantly lower content of protein, and a higher content of total fat and saturates in white GF bread products compared to their counterparts [31]. Our findings are in agreement with all these results, but not with Hopkins et al. [32], who reported only a lower protein content, and with Allen et al. [33], who found only higher fat and sugars, when GF bread products were compared with their counterparts.

In the Australian market, Wu et al. [34] only found a higher protein content in 54 GF bread items compared to the 483 gluten-containing counterparts, in line with the above-mentioned studies. Regarding the Italian market, the study of Cornicelli et al. [27] showed that 42 GF bread products had lower energy, total carbohydrate and protein contents than 42 regular counterparts. Among bread substitutes, GF items (*n* = 14) had a lower protein content than regular items (*n* = 36), but the latter had also a lower total carbohydrate content [27], in agreement with the present survey. Finally, a previous study of our group found a lower apparent nutritional quality in GF bread products (*n* = 24) compared to the regular counterparts (*n* = 34), but not between bread substitute categories [35]. These contrasting results might be explained by considering that, to compensate for gluten absence, GF products are generally made by using different mixtures of GF flours, fiber, hydrocolloids and enzymes, which deeply impact the nutritional profile [33].

When comparing products carrying or not NHC, the data highlighted a better nutritional profile in the former, above all for bread substitutes and for products with NC, for instance with on average a ~35% lower salt content in products boasting a NC compared to products without these claims. These results are partially comparable and in agreement with those of studies performed in the UK showing a better nutrition profile in different food categories boasting claims compared to products without them [36,37], despite other studies evidencing that the presence of a NHC is not necessarily related to an overall better nutrition profile [22]. Comparisons between products boasting or not NHCs have also been shown in previous studies within the FLIP project on different cereal-based products [25,38], finding contrasting results. Thus, overall the current evidence suggests that these claims should not be considered as markers of overall quality for all the food groups.

The major strength of the present study is attributable to the large number of the considered products, also the numerous online stores screened, which allowed the retrieval of data from complete and realistic pictures of the food pack items. At the same time, the consideration of only the online stores, and no other regular stores, such as discount or local shops, could have limited the number of the included products because some items (e.g., regional or artisanal products) could be not considered. Another limit of the study is the non-consideration of unpacked bread, since the nutrition declaration on the food pack is not mandatory [16], although this is still largely consumed in Italy. Finally, it would be of great interest to evaluate the nutritional quality of the products by considering the recommended/suggested portion, above all for bread substitutes which are often sold as single serving size. However, in accordance with Council Regulation (EU) 1169/2011, in Europe the nutrition declaration is mandatory only per 100 g of product, and not per serving, despite the fact that the energy value and the amounts of nutrients may be expressed per portion and/or per consumption unit, provided that the portion considered is quantified on the label. Moreover, when present, the suggested serving size is often different among products of the same category. This supports the importance of harmonizing serving size, which would allow them to provide clear nutrition information on the food pack and to help the consumer in making informed food choices [39]. Lastly, the analysis of fiber content in bread and substitutes would have been of interest, considering the growing interest in fiber of the research community and the consumers [40,41]. However, its declaration not being mandatory on nutritional labels in accordance with the above-mentioned Reg. (EU) 1169/2011, this aspect was not investigated.

To conclude, the present findings demonstrate the wide choice of bread products and bread substitutes on the market, with high variability in terms of ingredients and nutritional profile, and also confirmed the previous ones obtained by analyzing other food categories of products sold in Italy within the FLIP project. Moreover, the wide differences among the nutrition declarations of bread products and substitutes suggest that bread alternatives should not be considered *tout court* as substitutes from a nutritional point of view. Moreover, bread substitutes should not be considered as a unique category due to the wide differences in their energy and nutrient contents. For this reason, the consumer should be able to read the food labels and understand the carried information, in order to make informed and possibly healthy choices. Consequently, the introduction of Regulations for the mandatory presence of some information on the food pack is just one side of the coin, and should be combined with an effort to educate the consumer in reading and understanding the food labels during the shop.

## Figures and Tables

**Table 1:** Energy, macronutrients and salt across bread categories.

Items		Number of Items	Energy kcal/100 g	Fats		Carbohydrates		Protein g/100 g	Salt g/100 g
				Total g/100 g	Saturates g/100 g	Total g/100 g	Sugars g/100 g		
Category	Bread	339	274 (255–289)	4.4 (3.2–5.5)	0.7 (0.5–1.0)	47.4 (43.0–51.0)	4.5 (3.0–6.2)	8.5 (7.9–9.5)	1.3 (1.1–1.4)
Type	Loaf	101	265 (242–355) ^b^	2.5 (1.3–5.3) ^c^	0.4 (0.2–1.0) ^c^	48.5 (41.8–62.0) ^ab^	2.6 (1.7–3.9) ^c^	8.5 (6.4–11.0)	1.2 (1.0–1.5)
	Rolls	70	287 (276–302) ^a^	5.3 (4.0–6.1) ^a^	1.0 (0.8–1.7) ^a^	50.0 (48.4–51.0) ^a^	7.0 (4.5–7.5) ^a^	8.5 (8.1–8.8)	1.3 (1.2–1.4)
	Sliced bread	168	268 (256–281) ^b^	4.6 (3.6–5.4) ^b^	0.7 (0.6–1.0) ^b^	45.9 (42.9–49.0) ^b^	4.8 (3.5–5.9) ^b^	8.5 (8.0–9.2)	1.3 (1.2–1.4)
Gluten free	No	306	276 (257–290) ^a^	4.3 (3.2–5.5) ^b^	0.7 (0.5–1.0) ^b^	48.0 (44.0–51.0) ^a^	4.7 (3.1–6.2) ^a^	8.5 (8.1–9.6) ^a^	1.3 (1.2–1.4)
	Yes	33	261 (239–272) ^b^	6.2 (4.1–8.5) ^a^	1.0 (0.6–1.5) ^a^	43.0 (40.9–47.0) ^b^	3.3 (2.5–4.4) ^b^	3.9 (3.4–4.4) ^b^	1.2 (1.0–1.4)
Nutrition claim	No	248	276 (261–291) ^a^	4.6 (3.5–5.5)	0.7 (0.5–1.1) ^a^	49.0 (45.2–51.0) ^a^	4.7 (3.3–6.6) ^a^	8.5 (8.0–9.3)	1.3 (1.2–1.4) ^a^
	Yes	91	259 (242–279) ^b^	4.3 (1.4–5.8)	0.6 (0.3–1.0) ^b^	43.0 (39.0–47.0) ^b^	3.5 (2.4–5.5) ^b^	8.5 (5.7–10.0)	1.2 (1.0–1.4) ^b^
Health Claim	No	339	274 (255–289)	4.4 (3.2–5.5)	0.7 (0.5–1.0)	47.4 (43.0–51.0)	4.5 (3.0–6.2)	8.5 (7.9–9.5)	1.3 (1.1–1.4)
	Yes	0	n.a.	n.a.	n.a.	n.a.	n.a.	n.a.	n.a.

Values are expressed as median (25th–75th percentile). For each category item, different lowercase letters in the same column indicate significant difference (Kruskal–Wallis non-parametric one-way ANOVA for independent samples with multiple pairwise comparisons; Mann–Whitney non-parametric test for two independent samples), *p* < 0.05. Legend: n.a., not applicable.

**Table 2:** Energy, macronutrients and salt across bread substitute categories.

Items		Number of Items	Energy kcal/100 g	Fats		Carbohydrates		Protein g/100 g	Salt g/100 g
				Total g/100 g	Saturates g/100 g	Total g/100 g	Sugars g/100 g		
Category	Bread substitutes	1020	412 (380–437)	9.6 (6.0–13.0)	1.6 (0.9–2.8)	68.0 (62.8–73.0)	2.0 (1.2–3.2)	10.0 (8.2–12.0)	1.7 (1.0–2.2)
Type	Crackers	186	433 (415–442) ^b^	12.0 (9.6–13.0) ^b^	1.7 (1.2–2.1) ^b^	68.0 (64.4–72.0) ^bc^	2.1 (1.9–2.6) ^c^	10.2 (10.0–11.6) ^c^	1.9 (1.4–2.3) ^bc^
	Wraps	146	311 (304–333) ^d^	9.4 (8.0–11.2) ^c^	1.7 (1.3–3.9) ^ab^	48.3 (46.0–50.1) ^e^	1.3 (1.1–1.8) ^d^	7.7 (7.1–8.1) ^e^	1.6 (1.5–2.0) ^c^
	Breadsticks	197	419 (407–432) ^b^	9.7 (7.3–12.0) ^c^	1.8 (1.2–3.0) ^b^	69.9 (66.0–72.0) ^b^	2.5 (1.9–3.2) ^bc^	12.0 (11.0–13.0) ^a^	2.0 (1.8–2.3) ^ab^
	Rice and corn cakes	174	383 (376–393) ^c^	2.3 (1.5–3.7) ^e^	0.6 (0.4–1.0) ^c^	78.9 (72.0–82.0) ^a^	0.6 (0.4–0.9) ^e^	8.2 (7.4–11.0) ^d^	0.5 (0.1–1.0) ^e^
	Taralli	100	470 (458–479) ^a^	18.0 (17.0–20.0) ^a^	2.9 (2.0–3.2) ^a^	64.8 (63.0–67.0) ^d^	1.5 (1.1–1.9) ^d^	9.4 (8.7–10.0) ^d^	2.3 (2.1–2.5) ^a^
	Croutons, Bruschetta and “Frisella” bread	100	429 (402–4531) ^b^	11.8 (6.7–17.0) ^bc^	1.8 (1.0–3.4) ^b^	67.8 (62.0–71.4) ^cd^	3.2 (2.0–4.0) ^b^	11.0 (10.0–12.0) ^bc^	2.0 (1.5–2.5) ^ab^
	Rusks	117	401 (391–411) ^c^	6.8 (5.7–7.8) ^d^	1.0 (0.8–2.0) ^c^	72.0 (67.0–73.3) ^b^	6.5 (4.5–8.0) ^a^	11.1 (11.0–13.0) ^ab^	1.2 (0.8–1.5) ^d^
Gluten free	No	859	415 (384–439) ^a^	10.0 (7.1–13.3) ^a^	1.7 (1.0–3.0) ^a^	66.7 (61.5–71.0) ^b^	2.1 (1.5–3.5) ^a^	10.9 (9.3–12.0) ^a^	1.8 (1.3–2.2) ^a^
	Yes	161	386 (378–417) ^b^	2.3 (1.5–8.3) ^b^	0.6 (0.4–1.3) ^b^	80.0 (75.4–83.0) ^a^	0.6 (0.5–1.0) ^b^	7.5 (6.3–8.4) ^b^	0.5 (0.3–1.2) ^b^
Nutrition claim	No	664	421 (382–445) ^a^	11.0 (7.9–15.0) ^a^	1.9 (1.2–3.3) ^a^	67.0 (60.0–72.0) ^b^	2.0 (1.4–3.3) ^a^	10.0 (8.1–11.4) ^b^	1.9 (1.4–2.3) ^a^
	Yes	356	393 (379–417) ^b^	6.3 (2.4–10.0) ^b^	1.0 (0.6–1.6) ^b^	70.0 (65.0–77.9) ^a^	1.9 (0.7–3.0) ^b^	11.0 (8.6–12.8) ^a^	1.2 (0.5–1.8) ^b^
Health Claim	No	968	412 (379–437)	9.6 (6.1–13.0) ^a^	1.6 (0.9–2.9) ^a^	68.0 (62.8–73.0)	2.0 (1.2–3.2)	10.0 (8.2–11.9) ^b^	1.7 (1.1–2.2)
	Yes	52	412 (390–422)	8.0 (5.5–11.0) ^b^	1.2 (0.7–1.8) ^b^	69.3 (64.5–71.0)	2.0 (1.4–3.2)	12.3 (10.6–14.0) ^a^	1.5 (0.3–2.1)

Values are expressed as median (25th–75th percentile). For each category item, different lowercase letters in the same column indicate significant difference (Kruskal–Wallis non-parametric one-way ANOVA for independent samples with multiple pairwise comparisons; Mann–Whitney non-parametric test for two independent samples), *p* < 0.05.

## Supplementary Materials

The following are available online at https://www.mdpi.com/2304-8158/9/12/1905/s1, Figure S1: Principal component analysis (PCA) describing the inter-product variability among breads based on the nutritional composition of analyzed products (energy (kcal/100 g), total fat (g/100 g), saturates (g/100), carbohydrate (g/100 g), sugars (g/100 g), protein (g/100 g), and salt (g/100 g)). Loading plots of PC1 versus PC2 (IA), PC1 versus PC3 (IIA) and PC3 versus PC2 (IIIA); score plots of the nutrition composition for bread of PC1 versus PC2 (IB), PC1 versus PC3 (IIB) and PC3 versus PC2 (IIIB). Legend: A, loaf; B, rolls; C, sliced bread, Figure S2: Principal component analysis (PCA) describing the inter-product variability among bread substitutes based on the nutritional composition of analyzed products (energy (kcal/100 g), total fat (g/100 g), saturates (g/100), carbohydrate (g/100 g), sugars (g/100 g), protein (g/100 g), and salt (g/100 g)). Loading plots of PC1 versus PC2 (IA), PC1 versus PC3 (IIA) and PC3 versus PC2 (IIIA); score plots of the nutrition composition for bread substitutes of PC1 versus PC2 (IB), PC1 versus PC3 (IIB) and PC3 versus PC2 (IIIB). Legend: D, crackers; E, wraps; F, breadsticks; G, rice and corn cakes; H, taralli; I, croutons, bruschetta and “Frisella” bread; J, rusks.
