# Supplementary material for: Analysis of Food Labels to Evaluate the Nutritional Quality of Bread Products and Substitutes Sold in Italy: Results from the Food Labelling of Italian Products (FLIP) Study
Source: Foods. 2020 Dec 20;9(12):1905. doi: 10.3390/foods9121905 (PMC7766686; doi:10.3390/foods9121905)

SUPPLEMENTARY FIGURES

Supplementary Figure S1. Principal component analysis (PCA) describing the inter-product variability among breads based on the nutritional composition of analyzed products (energy (kcal/100 g), total fat (g/100 g), saturates (g/100), carbohydrate (g/100 g), sugars (g/100 g), protein (g/100 g), and salt (g/100 g)). Loading plots of PC1 versus PC2 (IA), PC1 versus PC3 (IIA) and PC3 versus PC2 (IIIA); score plots of the nutrition composition for bread of PC1 versus PC2 (IB), PC1 versus PC3 (IIB) and PC3 versus PC2 (IIIB). Legend: A, loaf; B, rolls; C, sliced bread.

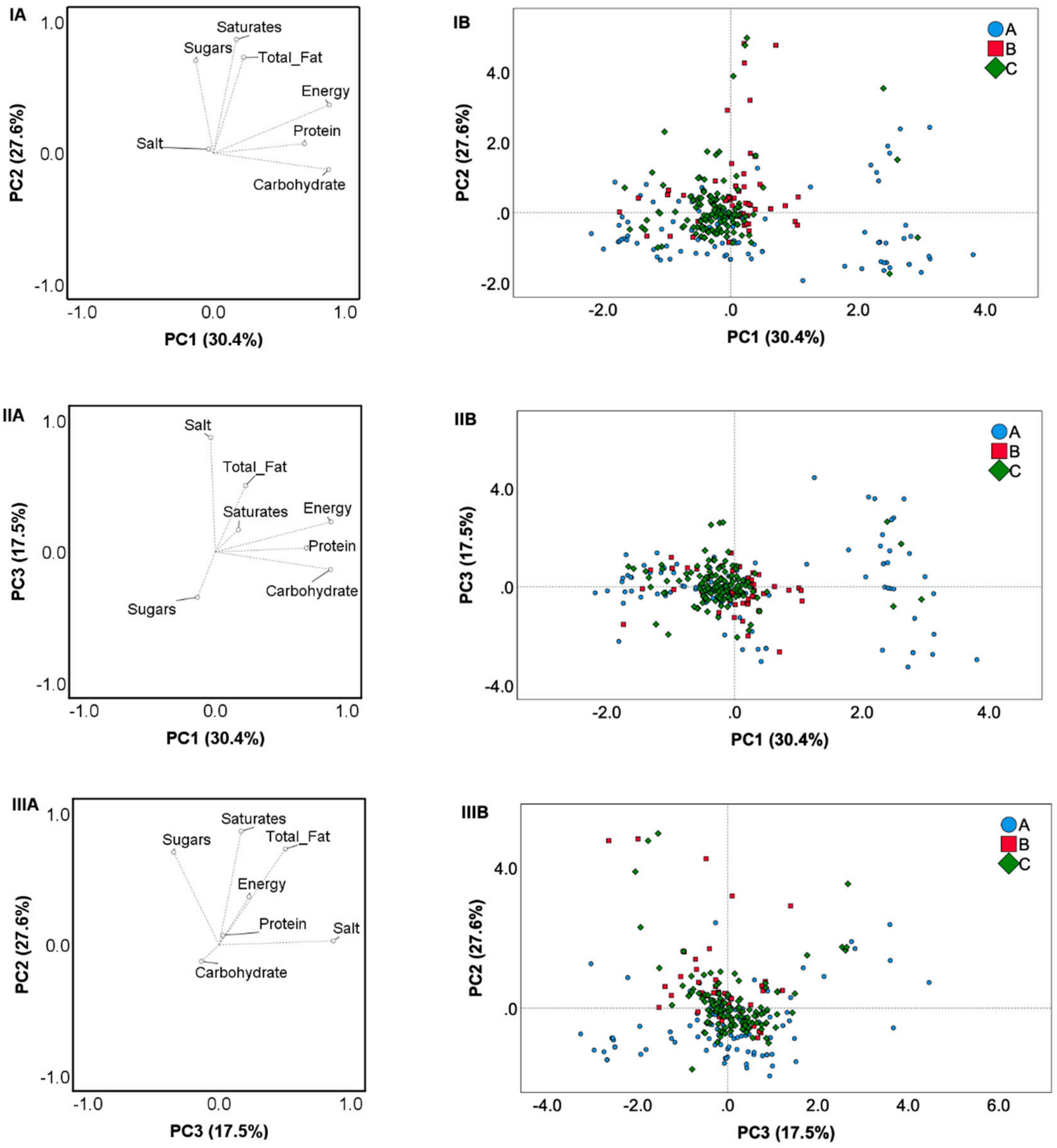

Supplementary Figure S2. Principal component analysis (PCA) describing the inter-product variability among bread substitutes based on the nutritional composition of analyzed products (energy (kcal/100 g), total fat (g/100 g), saturates (g/100), carbohydrate (g/100 g), sugars (g/100 g), protein (g/100 g), and salt (g/100 g)). Loading plots of PC1 versus PC2 (IA), PC1 versus PC3 (IIA) and PC3 versus PC2 (IIIA); score plots of the nutrition composition for bread substitutes of PC1 versus PC2 (IB), PC1 versus PC3 (IIB) and PC3 versus PC2 (IIIB). Legend: D, crackers; E, wraps; F, breadsticks; G, rice and corn cakes; H, *taralli*; I, croutons, bruschetta and "Frisella" bread; J, rusks.

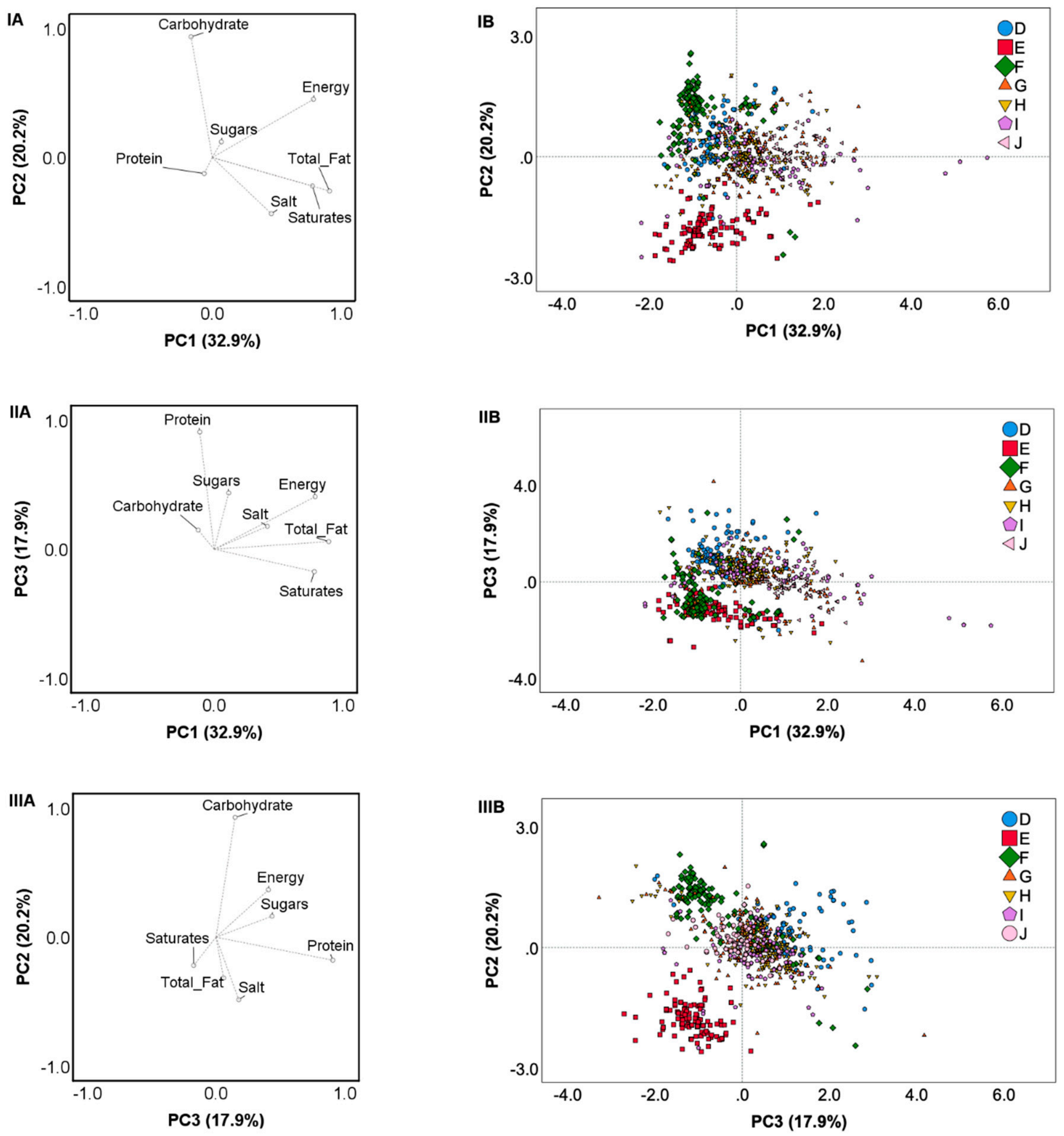

Supplement: Supplementary file 1 [file foods-09-01905-s001.pdf]
